# One kind hybrid character sums and their upper bound estimates

**DOI:** 10.1186/s13660-018-1753-4

**Published:** 2018-07-04

**Authors:** Jianhong Zhao, Xiao Wang

**Affiliations:** 1 Department of Teachers Education, Lijiang Teachers College, Lijiang, P.R. China; 2 School of Mathematics, Northwest University, Xi’an, P.R. China

**Keywords:** 11T24, The hybrid character sums, Lucas polynomials, Upper bound estimate, Gauss sums, Combinatorial identity

## Abstract

The main purpose of this paper is applying the analysis method, the properties of Lucas polynomials and Gauss sums to study the estimation problems of some kind hybrid character sums. In the end, we obtain several sharp upper bound estimates for them. As some applications, we prove some new and interesting combinatorial identities.

## Introduction

As usual, let $q\geq3$ be an integer, *χ* denotes any Dirichlet character $\bmod\, q$. The classical Gauss sum $\tau(\chi)$ is defined by

$$\tau(\chi)=\sum_{a=1}^{q}\chi(a)e \biggl( \frac{a}{q} \biggr), $$

where $e(y)=e^{2\pi i y}$.

We know that this sum plays a very important role in analytic number theory; plenty of number theory problems (such as Dirichlet *L*-functions and distribution of primes) are closely related to it. Concerning the various elementary properties of $\tau(\chi)$, some authors also studied it and obtained a series of interesting results, some conclusions can be found in Refs. [1] and [2]. For example, if *χ* is a primitive character $\bmod\, q$, then, for any integer *n*, one has $|\tau(\chi)|=\sqrt{q}$ and the identity

$$\sum_{a=1}^{q}\chi(a)e \biggl( \frac{na}{q} \biggr)=\overline{\chi}(n)\tau (\chi). $$

From the Euler formula we know that $e(x)=\cos(2\pi x)+i\sin(2\pi x)$. So scholars will naturally ask, for any positive integer *n*, whether there exists a similar estimate for

1 $$ \Biggl\vert \sum_{a=1}^{q} \chi(a)\cos^{n} \biggl(\frac{2\pi a}{q} \biggr) \Biggr\vert \quad \text{and}\quad \Biggl\vert \sum_{a=1}^{q} \chi(a)\sin^{n} \biggl(\frac{2\pi a}{q} \biggr) \Biggr\vert . $$

The estimates or calculations for (1) are significant, because they are closely related to the famous Gauss sums, so they have many interesting applications in analytic number theory, especially various estimates for hybrid character sums and generalized Kloosterman sums.

As far as we know, it seems that nobody has studied the estimate for (1), at least we have not seen related papers before. In this paper, we shall use the analytic method, the properties of Lucas polynomials and Gauss sums to do research on these problems, and obtain some sharp upper bound estimates for them. Our main idea is to put the *n*th power $\sin^{n} (x)$ (or $\cos^{n}(x)$) into a combination of $\sin(kx)$ (or $\cos (kx)$), where $1\leq k\leq n$. Then using the estimate for classical Gauss sums we give some sharp upper bound estimates for (1). As some applications of our main results, we also give some new and interesting combinatorial identities.

## Main results and discussion

The main results in this paper are detailed in the following.

### Theorem 1

*If* *q* *is an integer with* $q> 2$, *then*, *for any positive integer* *n* *and primitive character* $\chi\bmod\, q$, *we have the estimate*

$$\begin{aligned} (\mathrm{a})&\quad \Biggl\vert \sum_{a=1}^{q-1} \chi(a)\cos^{2n} \biggl(\frac{2\pi a}{q} \biggr) \Biggr\vert \leq \biggl(1-\frac{\binom{2n}{n}}{4^{n}} \biggr) \sqrt {q}; \\ (\mathrm{b})&\quad \Biggl\vert \sum_{a=1}^{q-1} \chi(a)\cos^{2n-1} \biggl(\frac{2\pi a}{q} \biggr) \Biggr\vert \leq \sqrt{q}, \end{aligned}$$

*where* $\binom{m}{n}=\frac{m!}{n!(m-n)!}$.

### Theorem 2

*If* *q* *is an integer with* $q> 2$, *then*, *for any positive integer* *n* *and primitive character* $\chi\bmod\, q$, *we have the estimate*

$$\begin{aligned} (\mathrm{c})&\quad \Biggl\vert \sum_{a=1}^{q-1} \chi(a)\sin^{2n} \biggl(\frac{2\pi a}{q} \biggr) \Biggr\vert \leq \biggl(1-\frac{\binom{2n}{n}}{4^{n}} \biggr) \sqrt {q}; \\ (\mathrm{d})&\quad \Biggl\vert \sum_{a=1}^{q-1} \chi(a)\sin^{2n-1} \biggl(\frac{2\pi a}{q} \biggr) \Biggr\vert \leq \sqrt{q}. \end{aligned}$$

From the method of proving Theorem 1 and the orthogonality of characters $\bmod\, q$ we can immediately deduce the following combinatorial identity.

### Corollary

*If* *n* *is any positive integer*, *then we have the identity*

$$\binom{4n}{2n} + \binom{2n}{n}^{2}= 2\sum _{k=0}^{n}\binom{2n}{k}^{2}. $$

### Notes

Note that $2\cos^{2} x= \cos(2x)+1$ and $|\tau(\chi)|=\sqrt{q}$; we have

$$\begin{aligned} \begin{aligned} \Biggl\vert \sum_{a=1}^{q-1}\chi(a) \cos^{2} \biggl(\frac{2\pi a}{q} \biggr) \Biggr\vert &= \frac{1}{2} \Biggl\vert \sum_{a=1}^{q-1} \chi(a)\cos \biggl(\frac{4\pi a}{q} \biggr)+\sum_{a=1}^{q-1} \chi(a) \Biggr\vert \\ &= \frac{1}{4} \Biggl\vert \sum_{a=1}^{q-1} \chi(a)e \biggl(\frac{2 a}{q} \biggr)+\sum_{a=1}^{q-1} \chi(a)e \biggl(\frac{-2 a}{q} \biggr) \Biggr\vert \\ &=\frac {1}{4}\sqrt{q} \bigl\vert \overline{\chi}(2)+\overline{ \chi}(-2) \bigr\vert \\ &= \textstyle\begin{cases} \frac{1}{2}\cdot\sqrt{q}, & \text{if }\chi(-1)=1, \\ 0, & \text{if }\chi(-1)=-1. \end{cases}\displaystyle \end{aligned} \end{aligned}$$

Therefore, if $n=1$ and *χ* is an even primitive character $\bmod\, q$, then the equal sign in our theorems holds. So the estimates in our Theorem 1 and Theorem 2 are the best.

## Several simple lemmas

In order to prove our main results, we first introduce the Fibonacci polynomials and Lucas polynomials as follows.

For integer $n\geq0$, the famous Fibonacci polynomials $\{F_{n}(x)\}$ and Lucas polynomials $\{L_{n}(x)\}$ are defined by $F_{0}(x)=0$, $F_{1}(x)=1$, $L_{0}(x)=2$, $L_{1}(x)=x$ and $F_{n+2}(x)=xF_{n+1}(x)+F_{n}(x)$, $L_{n+2}(x)=xL_{n+1}(x)+L_{n}(x)$ for all $n\geq0$. In fact the general terms of $F_{n}(x)$ and $L_{n}(x)$ are given by

$$F_{n}(x)=\frac{1}{\sqrt{x^{2}+4}} \biggl[ \biggl(\frac{x+\sqrt{x^{2}+4}}{2} \biggr)^{n}- \biggl(\frac{x-\sqrt{x^{2}+4}}{2} \biggr)^{n} \biggr] $$

and

2 $$ L_{n}(x)= \biggl(\frac{x+\sqrt{x^{2}+4}}{2} \biggr)^{n}+ \biggl(\frac{x-\sqrt {x^{2}+4}}{2} \biggr)^{n}. $$

It is easy to obtain the identities

$$F_{n+1}(x)=\sum_{k=0}^{ [\frac{n}{2} ]} \binom{n-k}{k}x^{n-2k} \quad \text{and}\quad L_{n}(x)= \sum _{k=0}^{ [\frac{n}{2} ]}\frac{n}{n-k} \binom{n-k}{k}x^{n-2k}, $$

where $\binom{m}{n}=\frac{m!}{n!(m-n)!}$, and $[x]$ denotes the greatest integer ≤*x*.

Taking $x=1$, then $\{F_{n}(x)\}$ becomes the Fibonacci sequences $\{F_{n}\}$, and $\{L_{n}(x)\}$ becomes the Lucas sequences $\{L_{n}\}$. If we take $x=2$, then $F_{n}(2)=P_{n}$, the *n*th Pell numbers, $P_{0}=0$, $P_{1}=1$, and $P_{n+2}=2P_{n+1}+P_{n}$ for all $n\geq0$.

Since these sequences (or polynomials) occupy a more crucial position in the theory and application of mathematics, many scholars have studied their various elementary properties and obtained a series of important results. See Refs. [3–10]. Here we give some new properties of Lucas polynomials.

### Lemma 1

*If* *k* *is a non*-*negative integer*, *then we have the identities*

$$\begin{aligned}& L_{2k} (2i\sin\theta )=2\cdot\cos (2k\theta ) \quad \textit{and}\quad L_{2k} (2i\cos\theta )=(-1)^{k}\cdot 2\cdot\cos (2k\theta ); \\& L_{2k+1} (2i\sin\theta )= 2i \cdot\sin\bigl((2k+1)\theta\bigr), \end{aligned}$$

*and*

$$L_{2k+1} (2i\cos\theta )= (-1)^{k}\cdot2i \cdot\cos \bigl((2k+1)\theta\bigr), $$

*where* *i* *is the imaginary unit*. *That is to say*, $i^{2}=-1$.

### Proof

Taking $x=2i\sin\theta$ in (2), and note that $x^{2}+4=4-4\sin^{2} \theta=4\cos^{2}\theta$, from the Euler formula we have

$$\begin{aligned} L_{2k} (2i\sin\theta ) =& \biggl(\frac{2i\sin\theta+ \sqrt{4\cos ^{2}\theta}}{2} \biggr)^{2k}+ \biggl(\frac{2i\sin\theta- \sqrt{4\cos^{2}\theta }}{2} \biggr)^{2k} \\ =& (i\sin\theta+\cos\theta )^{2k}+ (i\sin\theta-\cos \theta )^{2k} \\ =& (\cos\theta+i\sin\theta )^{2k}+ (\cos\theta-i\sin \theta )^{2k} \\ =&\cos(2k\theta)+i\sin(2k\theta)+\cos(2k\theta)-i\sin(2k\theta)=2 \cdot\cos(2k \theta) \end{aligned}$$

and

$$\begin{aligned} L_{2k} (2i\cos\theta ) =& \biggl(\frac{2i\cos\theta+ \sqrt{4\sin ^{2}\theta}}{2} \biggr)^{2k}+ \biggl(\frac{2i\cos\theta- \sqrt{4\sin^{2}\theta }}{2} \biggr)^{2k} \\ =& (i\cos\theta+\sin\theta )^{2k}+ (i\cos\theta-\sin \theta )^{2k} \\ =&(-1)^{k} (\cos\theta-i\sin\theta )^{2k}+(-1)^{k} (\cos \theta+i\sin\theta )^{2k} \\ =&(-1)^{k} \cdot2\cdot\cos(2k\theta). \end{aligned}$$

This proves the first and second formulas of Lemma 1.

Similarly, we also have the identities

$$\begin{aligned} L_{2k+1} (2i\sin\theta ) =& \biggl(\frac{2i\sin\theta+ \sqrt {4\cos^{2}\theta}}{2} \biggr)^{2k+1}+ \biggl(\frac{2i\sin\theta- \sqrt{4\cos ^{2}\theta}}{2} \biggr)^{2k+1} \\ =& (i\sin\theta+\cos\theta )^{2k+1}+ (i\sin\theta-\cos \theta )^{2k+1} \\ =& (\cos\theta+i\sin\theta )^{2k+1}- (\cos\theta -i\sin\theta )^{2k+1} \\ =&\cos\bigl((2k+1)\theta\bigr)+i\sin\bigl((2k+1)\theta\bigr)-\cos\bigl((2k+1) \theta\bigr)+i\sin \bigl((2k+1)\theta\bigr) \\ =& 2i \cdot\sin\bigl((2k+1)\theta\bigr) \end{aligned}$$

and

$$\begin{aligned} L_{2k+1} (2i\cos\theta ) =& \biggl(\frac{2i\cos\theta+ \sqrt {4\sin^{2}\theta}}{2} \biggr)^{2k+1}+ \biggl(\frac{2i\cos\theta- \sqrt{4\sin ^{2}\theta}}{2} \biggr)^{2k+1} \\ =& (i\cos\theta+\sin\theta )^{2k+1}+ (i\cos\theta-\sin \theta )^{2k+1} \\ =&i^{2k+1} (\cos\theta-i\sin\theta )^{2k+1}+i^{2k+1} ( \cos\theta+i\sin\theta )^{2k+1} \\ =& (-1)^{k}\cdot2i \cdot\cos\bigl((2k+1)\theta\bigr). \end{aligned}$$

This completes the proof of Lemma 1. □

### Lemma 2

*If* *n* *is any non*-*negative integer*, *then we have the identities*

$$ x^{2n}= \frac{(-1)^{n}}{2}\binom{2n}{n}\cdot L_{0}(x)+ \sum_{k=1}^{n}(-1)^{n-k} \binom{2n}{n-k}\cdot L_{2k}(x) $$

*and*

$$ x^{2n+1}= \sum_{k=0}^{n}(-1)^{n-k} \binom{2n+1}{n-k}\cdot L_{2k+1}(x). $$

### Proof

From the definition of $L_{n}(x)$ we know that $L_{2k}(x)$ is an even function. So we may suppose that

3 $$ x^{2n}= \sum_{k=0}^{n} a_{k} \cdot L_{2k}(x). $$

Taking $x=2i\cos\theta$ in (3) and applying Lemma 1, we have

4 $$ (-1)^{n}4^{n}\cos^{2n} \theta= \sum _{k=0}^{n} a_{k}\cdot L_{2k} (2i\cos \theta )=2\sum_{k=0}^{n} a_{k}\cdot(-1)^{k}\cos (2k\theta ). $$

Note that the identities

5 $$ \int_{0}^{\pi}2\cos(m\theta)\cos(n\theta)\,d\theta= \textstyle\begin{cases} \pi, &\text{if }m=n\neq0, \\ 0, &\text{if } m\neq n, \\ 2\pi, &\text{if }m= n=0, \end{cases} $$

and

$$\int_{0}^{\pi}\cos^{2n} (\theta)\cos (2k \theta )\,d\theta=\pi\cdot \frac{(2n)!}{(2n-2k)!!(2n+2k)!!}=\frac{\pi}{4^{n}}\cdot \binom{2n}{n-k}, $$

from (4) we have

$$ a_{k}\cdot(-1)^{k} \pi=(-1)^{n} 4^{n} \int_{0}^{\pi}\cos^{2n} (\theta)\cos (2k \theta )\,d\theta=(-1)^{n}\pi\cdot\binom{2n}{n-k} $$

or

6 $$ a_{0}=\frac{(-1)^{n}}{2} \cdot\binom{2n}{n} \quad \text{and}\quad a_{k}=(-1)^{n-k} \cdot\binom{2n}{n-k}, \quad 1\leq k\leq n. $$

Combining identities (3) and (6) we may immediately deduce the first formula of Lemma 2.

Similarly, since $L_{2k+1}(x)$ is an odd function, we can suppose that

7 $$ x^{2n+1}= \sum_{k=0}^{n} b_{k} \cdot L_{2k+1}(x). $$

Taking $x=2i\cos\theta$ in (7), then applying Lemma 1 we have

8 $$ (-1)^{n} 4^{n} \cos^{2n+1} \theta= \sum_{k=0}^{n} b_{k} \cdot(-1)^{k} \cos \bigl((2k+1)\theta \bigr). $$

From (5) and (8) we may immediately deduce that

9 $$ b_{k}= \frac{2(-1)^{n-k}}{\pi}\cdot4^{n}\cdot \int_{0}^{\pi}\cos^{2n+1} (\theta) \cos \bigl((2k+1)\theta \bigr)\,d\theta=(-1)^{n-k} \binom{2n+1}{n-k}. $$

Now the second identity of Lemma 2 follows from (7) and (9). □

### Lemma 3

*If* *n* *is a positive integer*, *then we have the identity*

$$\sum_{k=1}^{n}\binom{2n}{n-k}= \frac{1}{2}\cdot \biggl(4^{n}-\binom{2n}{n} \biggr)\quad \textit{and}\quad \sum_{k=0}^{n} \binom{2n+1}{n-k}=4^{n}. $$

### Proof

First applying the binomial theorem we have the identity

10 $$ \sum_{k=0}^{2n} \binom{2n}{2n-k}=\sum_{k=0}^{2n} \binom{2n}{k}=(1+1)^{2n}=4^{n}. $$

On the other hand, we also have

11 $$\begin{aligned} \sum_{k=0}^{2n}\binom{2n}{k}=(1+1)^{2n} =& \sum_{k=0}^{n}\binom{2n}{k}+ \sum _{k=n+1}^{2n}\binom{2n}{k} \\ =&\sum_{k=0}^{n}\binom{2n}{k}+ \sum _{k=1}^{n}\binom{2n}{n-k} \\ =& 2\sum _{k=1}^{n}\binom{2n}{n-k}+\binom{2n}{n}. \end{aligned}$$

Combining (10) and (11) we can deduce

$$\sum_{k=1}^{n}\binom{2n}{n-k}= \frac{1}{2}\cdot \biggl(4^{n}-\binom{2n}{n} \biggr). $$

This proves the first formula of Lemma 3.

Similarly, we can deduce the second formula of Lemma 3. □

## Proofs of the theorems

In this section, we complete the proofs of our main results. First we prove Theorem 1. Let $q\geq3$ be an integer; *χ* is any even primitive character $\bmod\, q$. Then taking $x=2i \cos (\frac{2 \pi a}{q} )$ in the first formula of Lemma 2, multiplying both sides by $\chi(a)$ and summing over all $1\leq a\leq q-1$, and noting that

$$\sum_{a=1}^{q-1}\chi(a)=0 \quad \text{and} \quad \sum_{a=1}^{q-1}\chi(a)e \biggl( \frac{\pm 2ka}{q} \biggr)=\overline{\chi}(\pm2k)\tau(\chi), $$

we have

12 $$\begin{aligned}& (-1)^{n} 4^{n}\sum_{a=1}^{q-1} \chi(a)\cos^{2n} \biggl(\frac{2\pi a}{q} \biggr) \\& \quad = (-1)^{n}\binom{2n}{n}\sum_{a=1}^{q-1} \chi(a)+ 2\sum_{k=1}^{n}(-1)^{n-k} \binom{2n}{n-k}\sum_{a=1}^{q-1}(-1)^{k} \chi(a)\cos \biggl(\frac{4k\pi a}{q} \biggr) \\& \quad = (-1)^{n}\sum_{k=1}^{n} \binom{2n}{n-k}\sum_{a=1}^{q-1}\chi(a) \biggl[e \biggl(\frac{2ka}{q} \biggr)+e \biggl(\frac{-2ka}{q} \biggr) \biggr] \\& \quad = (-1)^{n}\tau(\chi)\sum_{k=1}^{n} \binom{2n}{n-k} \bigl(\overline{\chi }(2k)+\overline{\chi}(-2k) \bigr) \\& \quad = 2(-1)^{n}\overline{\chi}(2)\tau(\chi)\sum _{k=1}^{n}\binom {2n}{n-k}\overline{ \chi}(k). \end{aligned}$$

Note that $|\tau(\chi)|=\sqrt{q}$, from (12) and Lemma 3 we have the estimate

13 $$\begin{aligned} \Biggl\vert \sum_{a=1}^{q-1}\chi(a) \cos^{2n} \biggl(\frac{2\pi a}{q} \biggr) \Biggr\vert =& \frac{2\sqrt{q}}{4^{n}}\cdot \Biggl\vert \sum_{k=1}^{n} \binom {2n}{n-k}\overline{\chi}(k) \Biggr\vert \\ \leq& \frac{2\sqrt{q}}{4^{n}}\cdot\sum_{k=1}^{n} \binom{2n}{n-k} \\ =& \frac{2\sqrt{q}}{4^{n}}\cdot\frac{4^{n}-\binom{2n}{n}}{2} \\ =& \biggl(1- \frac {\binom{2n}{n}}{4^{n}} \biggr)\cdot \sqrt{q}. \end{aligned}$$

Similarly, taking $x=2i \cos (\frac{2\pi a}{q} )$ in the second formula of Lemma 2, then applying Lemma 1 we also have

14 $$\begin{aligned}& (-1)^{n} 4^{n}\sum_{a=1}^{q-1} \chi(a)\cos^{2n+1} \biggl(\frac{2\pi a}{q} \biggr) \\& \quad = \sum_{k=0}^{n}(-1)^{n-k} \binom{2n+1}{n-k}\sum_{a=1}^{q-1}(-1)^{k} \chi (a)\cos \biggl(\frac{2(2k+1)\pi a}{q} \biggr) \\& \quad = \frac{(-1)^{n}}{2}\cdot\sum_{k=0}^{n} \binom{2n+1}{n-k}\sum_{a=1}^{q-1}\chi(a) \biggl[e \biggl(\frac{(2k+1)a}{q} \biggr)+e \biggl(\frac {-(2k+1)a}{q} \biggr) \biggr] \\& \quad = \frac{(-1)^{n}\tau(\chi)}{2}\cdot\sum_{k=0}^{n} \binom{2n+1}{n-k} \bigl(\overline{\chi}(2k+1)+\overline{\chi}(-2k-1) \bigr) \\& \quad = (-1)^{n}\tau(\chi)\sum_{k=0}^{n} \binom{2n+1}{n-k}\overline{\chi}(2k+1). \end{aligned}$$

From (14) and Lemma 3 we may immediately deduce the estimate

15 $$\begin{aligned} \begin{aligned}[b] \Biggl\vert \sum_{a=1}^{q-1}\chi(a) \cos^{2n+1} \biggl(\frac{2\pi a}{q} \biggr) \Biggr\vert &= \frac{\sqrt{q}}{4^{n}}\cdot \Biggl\vert \sum_{k=0}^{n} \binom{2n+1}{n-k}\overline {\chi}(2k+1) \Biggr\vert \\ &\leq\frac{\sqrt{q}}{4^{n}}\cdot \sum_{k=0}^{n} \binom{2n+1}{n-k}=\sqrt{q}. \end{aligned} \end{aligned}$$

If *χ* is an odd primitive character $\bmod\, q$, then it is very easy to prove that

16 $$ \Biggl\vert \sum_{a=1}^{q-1} \chi(a)\cos^{2n+1} \biggl(\frac{2\pi a}{q} \biggr) \Biggr\vert = \Biggl\vert \sum_{a=1}^{q-1}\chi(a) \cos^{2n} \biggl(\frac {2\pi a}{q} \biggr) \Biggr\vert =0. $$

Combining (13), (15), and (16) we may immediately deduce Theorem 1.

Using a very similar method to proving Theorem 1 we can also deduce the estimates in Theorem 2. So it is not repeated here.

Now we prove our corollary. If *p* is a prime large enough, then, for any fixed integer $n\geq1$, from [11] we have the identity

17 $$ \sum_{a=0}^{p-1} \cos^{2n} \biggl(\frac{2\pi a}{p} \biggr)=\frac{p}{4^{n}}\cdot \binom{2n}{n}. $$

From the orthogonality of characters $\bmod\, p$ and (17) we have

18 $$\begin{aligned} \sum_{\chi\bmod\, p} \Biggl\vert \sum _{a=1}^{q-1}\chi(a)\cos^{2n} \biggl( \frac {2\pi a}{q} \biggr) \Biggr\vert ^{2} =&(p-1)\sum _{a=1}^{p-1}\cos^{4n} \biggl( \frac {2\pi a}{p} \biggr) \\ =&\frac{p(p-1)}{4^{2n}}\cdot\binom{4n}{2n}-(p-1). \end{aligned}$$

On the other hand, from (13), (17), and Lemma 3 we also have

19 $$\begin{aligned}& \sum_{\chi\bmod\, p} \Biggl\vert \sum _{a=1}^{p-1}\chi(a)\cos^{2n} \biggl( \frac {2\pi a}{p} \biggr) \Biggr\vert ^{2} \\& \quad = \Biggl\vert \sum_{a=1}^{p-1} \cos^{2n} \biggl(\frac{2\pi a}{p} \biggr) \Biggr\vert ^{2}+ \sum_{\substack{\chi\bmod\, p\\{\chi(-1)=1, \chi\neq\chi_{0} }}} \Biggl\vert \sum _{a=1}^{p-1}\chi(a)\cos^{2n} \biggl( \frac{2\pi a}{p} \biggr) \Biggr\vert ^{2} \\& \quad = \biggl( \frac{p}{4^{n}}\cdot\binom{2n}{n}-1 \biggr)^{2}+ \frac{4p}{4^{2n}} \sum_{\substack{\chi\bmod\, p\\{\chi(-1)=1, \chi\neq \chi_{0}}}} \Biggl\vert \sum_{k=1}^{n}\binom{2n}{n-k} \overline{\chi}(k) \Biggr\vert ^{2} \\& \quad = \biggl( \frac{p}{4^{n}}\cdot\binom{2n}{n}-1 \biggr)^{2}+ \frac {4p}{4^{2n}}\cdot\frac{p-1}{2}\sum_{k=1}^{n} \binom{2n}{n-k}^{2} -\frac {4p}{4^{2n}}\cdot \Biggl( \sum _{k=1}^{n}\binom{2n}{n-k} \Biggr)^{2} \\& \quad = \biggl( \frac{p}{4^{n}}\cdot\binom{2n}{n}-1 \biggr)^{2}+ \frac{4p}{4^{2n}}\cdot \frac{p-1}{2}\sum_{k=1}^{n} \binom{2n}{n-k}^{2}-\frac{p}{4^{2n}} \biggl(4^{n}- \binom{2n}{n} \biggr)^{2}. \end{aligned}$$

Combining (18) and (19) we have the identity

20 $$\begin{aligned} \frac{p(p-1)}{4^{2n}}\cdot\binom{4n}{2n}-(p-1) =& \biggl( \frac {p}{4^{n}} \cdot\binom{2n}{n}-1 \biggr)^{2}+ \frac{4p}{4^{2n}}\cdot \frac {p-1}{2}\sum_{k=1}^{n} \binom{2n}{n-k}^{2} \\ &{} -\frac{p}{4^{2n}} \biggl(4^{n}-\binom{2n}{n} \biggr)^{2}. \end{aligned}$$

From (20) we may immediately deduce

$$\binom{4n}{2n} = \binom{2n}{n}^{2}+ 2\sum _{k=1}^{n}\binom{2n}{n-k}^{2}=2\sum _{k=0}^{n}\binom{2n}{k}^{2} - \binom{2n}{n}^{2} . $$

This completes the proofs of our all results.
